# Beyond mitotic arrest: the diverse effects of microtubule-targeting drugs on tumor vasculature

**DOI:** 10.1038/s44321-025-00223-5

**Published:** 2025-03-26

**Authors:** April L Risinger

**Affiliations:** The University of Texas Health Science Center at San Antonio, Department of Pharmacology, San Antonio, Texas 78229 USA

**Keywords:** Cancer, Immunology, Vascular Biology & Angiogenesis

## Abstract

A. Risinger discusses the study by He et al, in this issue of *EMBO Mol Med*, that explores the potential of microtubule-binding drugs to affect angiogenic pericytes, and thereby the tumor microenvironment.

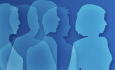

## Teaser

For decades microtubule-targeting agents (MTAs) have been a cornerstone of cancer treatment regimens and continue to be heavily employed as backbone chemotherapy in combination with new targeted agents. These drugs include both microtubule stabilizers (e.g. taxanes, ixabepilone) as well as destabilizers (e.g., vinca alkaloids, eribulin). MTAs are commonly referred to as antimitotic drugs based on the rationale that their anticancer efficacy is thought to be primarily due to the cell cycle arrest and subsequent apoptosis of rapidly dividing cancer cells downstream of microtubule disruption. However, accumulating evidence suggests that pharmacological microtubule disruption can also exert profound effects on the tumor microenvironment (TME), particularly through their influence on tumor vasculature and immune infiltration. While several MTAs with distinct binding sites and allosteric effects on microtubule structure are approved for the treatment of various cancers, little is known regarding how these drugs might differentially impact the TME. The recent study by He et al (2025) begins to shed light on these issues by demonstrating that structurally and functionally diverse MTAs can elicit markedly distinct effects on tumor vasculature through a process of pericyte phenotypic switching. These findings support the growing body of literature demonstrating that the antitumor efficacy of MTAs extends beyond their shared antimitotic effects and suggests these drugs could be used in a more rational and targeted manner, particularly as backbone chemotherapy in combination with immunotherapies and other targeted agents.

## Pericyte phenotype switching: a key driver of tumor vascular remodeling

Tumor vasculature is highly abnormal, characterized by disorganized, leaky blood vessels with poor perfusion and limited immune infiltration. One major contributor to this dysfunction is the behavior of pericytes, which support endothelial cells in normal blood vessels but become immature and dysregulated in tumors, contributing to vascular instability. He et al, show that eribulin as well as the investigational microtubule destabilizer combretastatin A-4 (CA-4) induce pericyte phenotype switching, promoting a transition from this dysfunctional state to a mature, contractile phenotype, which in turn stabilizes tumor blood vessels, improves perfusion, and reduces hypoxia. Crucially, this study also differentiates this effect of eribulin and CA-4 from other MTAs, such as taxanes and vinca alkaloids, which do not appear to have any significant effects on tumor vasculature. These findings align with prior work demonstrating the effects of eribulin on stabilization of the tumor vasculature. Funahashi et al, first demonstrated that eribulin induces vascular remodeling and increased perfusion in breast cancer xenograft models, which was sufficient to reverse tumor hypoxia and enhance drug delivery through signaling pathways that were speculated to regulate endothelial cell-pericyte interactions (Funahashi et al, 2014).

The critical importance of the microtubule cytoskeleton for the integrity of both normal and tumor vasculature and the ability of pharmacological microtubule disruption to promote RhoA-mediated changes to the endothelial network is well established (Bayless and Davis, 2004). However, the exact mechanisms and potential to utilize these observations to meaningfully improve the use of microtubule targeted drugs as anticancer agents is only now becoming clear. A guanine nucleotide exchange factor (GEF) that regulates RhoA-dependent cell contractility, GEF-H1, is sequestered on microtubules such that upon pharmacological microtubule depolymerization it is liberated to promote RhoA-dependent cytoskeletal reorganization and increased endothelial permeability (Chang et al, 2008). In contrast, microtubule-stabilizing taxanes do not allow the release of GEF-H1 providing one rationale for why microtubule stabilizers and destabilizers could have distinct effects on tumor vasculature. Intriguingly, the effects of eribulin and paclitaxel on the gene expression profile of pericytes in vitro were found to be profoundly distinct and in contrast to endothelial cells where the effects of the two drugs were largely overlapping (Agoulnik et al, 2014). Together these data provide additional support that microtubule destabilization by eribulin can modulate pericyte function at least in part through the release of GEF-H1 from microtubules to remodel tumor vasculature in a manner distinct from the taxane class of microtubule stabilizers.

## Colchicine-site agents: vascular disruptors or stabilizers? The importance of dose

A particularly striking finding of the current study is the dose-dependent vascular stabilizing effects of the colchicine site MTA, CA-4, since agents of this investigational drug class have been well-described as vascular disrupting agents that reduce blood flow to the tumor due to morphological changes to endothelial cells. Unlike eribulin, which consistently stabilizes tumor vasculature, and the taxanes and vinca alkaloids that do not significantly alter tumor vasculature over a range of concentrations, colchicine-site agents like CA-4 appear to exhibit varied effects depending on dose. At low doses, CA-4 stabilizes tumor vasculature and promotes immune infiltration (He et al, 2025), while at higher doses, it disrupts the vasculature, causing vessel collapse, hemorrhage, and tumor necrosis (Taguchi et al, 2021). Taken together, these studies suggest that careful dose modulation could allow colchicine-site MTAs to be used in vascular-stabilizing manner, rather than solely as vascular disruptors.

## The role of tumor vasculature in immune infiltration

One of the most important implications of the findings that MTAs have distinct effects on tumor vasculature is that a normalization of tumor vasculature would be expected to improve drug delivery as well as immune infiltration to provide long-lasting antitumor efficacy. Indeed, He et al, demonstrates that eribulin treatment results in significantly increased immune cell infiltration into tumors (He et al, 2025). This is consistent with prior reports demonstrating that eribulin can increase tumor microvessel density, improve tumor perfusion, and enhance immune infiltration (Funahashi et al, 2014; Goto et al, 2018; Ito et al, 2017). In particular, Ito et al, demonstrated that eribulin significantly increased microvessel density (MVD) and tumor perfusion that correlated with enhanced antitumor efficacy in multiple tumor models and was distinct from the effects of vinorelbine (Ito et al, 2017). This mechanistic difference between eribulin and vinorelbine is consistent with the current findings of He et al, but is somewhat surprising due to the fact that these agents are both microtubule destabilizing agents that bind within the vinca domain on microtubules albeit with distinct stoichiometry of binding and resulting effects on microtubule dynamicity that could underlie the phenotypic differences between these agents. Overall, it is clear that the distinct effects of MTAs on pericytes that differentially alter the tumor vasculature have clinically important consequences on the local tumor immune microenvironment. Most strikingly, the finding that selective depletion of natural killer (NK) cells is sufficient to diminish the antitumor efficacy of eribulin in multiple tumor models underscores the critical role of eribulin-mediated immune infiltration, which could actually be more important than the direct cytotoxic effects of the drug to cancer cells in some contexts (Hassouneh et al, 2024; Ito et al, 2017). This may also underlie the survival advantage observed for metastatic breast cancer patients treated with eribulin as compared to other chemotherapeutic drugs, including other MTAs (Cortes et al, 2011).

## The future of personalized chemotherapy

The mechanistic diversity of MTAs is often overlooked as these drugs have traditionally been viewed as non-selective cytotoxins. However, the findings from He et al, and prior literature indicate that MTAs could be used in a more targeted and personalized manner based at least in part on their distinct effects on tumor vasculature and immune infiltration. Understanding these distinctions could transform the way MTAs are used in cancer therapy. By shifting away from the outdated view of MTAs as one-size-fits-all cytotoxins, and instead leveraging their mechanistic diversity, we can design more rational, personalized chemotherapy regimens that optimize both direct tumoricidal activity and immune system engagement.
